# Learning Curve and Clinical Outcomes of Performing Surgery with the InterTan Intramedullary Nail in Treating Femoral Intertrochanteric Fractures

**DOI:** 10.1155/2017/6781070

**Published:** 2017-04-19

**Authors:** A-Bing Li, Wei-Jiang Zhang, Ji-Qi Wang, You-Ming Zhao, Wei-Jun Guo

**Affiliations:** Department of Orthopaedics, The Second Affiliated Hospital and Yuying Children's Hospital of Wenzhou Medical University, No. 109 Xue Yuan Xi Road, Wenzhou, Zhejiang 325000, China

## Abstract

*Purpose*. The purpose of this study is to evaluate the learning curve of performing surgery with the InterTan intramedullary nail in treating femoral intertrochanteric fractures, to provide valuable information and experience for surgeons who decide to learn a new procedure.* Methods*. We retrospectively analyzed data from 53 patients who underwent surgery using an InterTan intramedullary nail at our hospital between July 2012 and September 2015. The negative exponential curve-fit regression analysis was used to evaluate the learning curve. According to 90% learning milestone, patients were divided into two group, and the outcomes were compared.* Results*. The mean operative time was 69.28 (95% CI 64.57 to 74.00) minutes; with the accumulation of surgical experience, the operation time was gradually decreased. 90% of the potential improvement was expected after 18 cases. In terms of operative time, intraoperative blood loss, hospital stay, and Harris hip score significant differences were found between two groups (*p* = 0.009, *p* = 0.000, *p* = 0.030, and *p* = 0.002, resp.). Partial weight bearing time, fracture union time, tip apex distance, and the number of blood transfusions and complications were similar between two groups (*p* > 0.5).* Conclusion*. This study demonstrated that the learning curve of performing surgery with the InterTan intramedullary nail is acceptable and 90% of the expert's proficiency level is achieved at around 18 cases.

## 1. Introduction

Femoral intertrochanteric fractures are the second most common type of hip fracture. Both intramedullary and extramedullary internal fixation devices are widely used to treat femoral intertrochanteric fractures. The sliding hip screw (SHS) was regarded as the standard fixation device in care for femoral intertrochanteric fractures [1]. However, intramedullary nailing has improved biomechanical features compared to SHS, and many surgeons would likely select intramedullary devices for the treatment of femoral intertrochanteric fractures [2]. The TRIGEN INTERTAN Intertrochanteric Antegrade nail (Smith & Nephew, Memphis, TN) was introduced in 2005 and increases intertrochanteric rotational stability [3] and decreases lag screw cutout [4, 5].

The learning curve for orthopedic surgery [6, 7] and nonorthopedic surgeries [8, 9] has been previously described, but the learning curve of performing surgery with InterTan intramedullary nail (IT) has not been previously analyzed. Understanding the average learning curve for surgery with a specific device is in the best interests of patient safety and an important component of a surgeon's learning process [10]. Therefore, the aim of this study was to evaluate the learning curves of performing surgery with the InterTan intramedullary nail for treatment of femoral intertrochanteric fractures, to provide valuable information and experience for surgeons who decide to learn a new procedure.

## 2. Materials and Methods

### 2.1. Patients

We retrospectively analyzed data from 53 patients who underwent surgery using an InterTan intramedullary nail at our hospital between July 2012 and September 2015. Patients with femoral intertrochanteric fracture were considered eligible patients. Exclusion criteria were (i) age < 60 years, (ii) pathological fracture, (iii) old fracture or multiple fractures, (iv) an inability to walk or hemiplegia before the fracture, and (v) severe dementia or Parkinson's disease. All operations were performed by the same surgeon (Y.M.Z), who have more than 5 years' experience in DHS, and no experience with another cephalomedullary nail. The surgeon studied the operating instructions and the instructional video and performed a related exercise on the cadaver model, eventually operating on 10 patients with the help of an experienced surgeons before the operation of InterTan nail. Data were retrospectively collected from clinical records and outpatient follow-up for each patient. Official approval from the Investigational Ethical Review Board was waived by our hospital because of the retrospective design of the study. Informed consent that data could be used for research purposes was obtained from all participants or their authorized persons. Routine workup including routine blood test, blood biochemical analysis, electrocardiogram, blood coagulation function, X-ray, and other tests were collected as available. Physicians assisted in the treatment of varied medical conditions during the perioperative period. The patients received conventional postoperative intravenous injection of antibiotics for three days, and blood transfusion was performed in the case of Hb < 70 g/L. The anteroposterior and lateral position X-ray films of the hip joint in the affected side were reexamined 2 to 3 days after operation. The patient was instructed to conduct active contraction exercise on quadriceps femoris on the 2nd day after operation and gradually performed functional exercises in bed. The patient was encouraged to sit up within 1 week according to his/her conditions, conducted off-bed non-weight-bearing activities with assistance of waling aid 2 to 4 weeks after operation, began limited weight bearing activities with assistance of walking aid 5 to 8 weeks later, and completed weight bearing activities without assistance of waling aid after the clinical healing of the fracture.

### 2.2. Outcomes Measurements

Baseline characteristics included patient demographics, fracture classification, and American Society of Anesthesiology score. Study variables included operation time, intraoperative blood loss, weight bearing time, Harris hip score, length of hospital stay, fracture union time, tip apex distance (TDA), postoperative complications, and mortality. Harris hip scores were measured at one year after surgery. Operative time is defined as the interval from the incision of skin to the incision being closed. It does not include closed reduction time.

### 2.3. Surgical Technique

In this study, all patients were treated with the TRIGEN INTERTAN long nail (Smith & Nephew, USA), and the surgical procedure was performed according to the surgical technique specified by Smith & Nephew. Briefly, the surgical procedure was performed as follows: (1) Patient positioning: after general anesthesia or combined spinal epidural anesthesia, the patient was laid in the supine position on an extension table. Abduct the unaffected limb and place it on a foot holder. In order to facilitate nail access to the medullary cavity, adduct the affected limb by 10–15°. (2) Preparation: closed reduction of the fracture was performed under the monitor of “C” arm X-ray machine. After surgery area disinfection, shop sterile surgical towels, make a 4–6 cm longitudinal incision proximal from the tip of the greater trochanter. (3) Opening the proximal femur: A position slightly inside of the apex of the greater trochanter was used as the entry point at which the guide wire was inserted to the appropriate position. The correct positioning of the guide wire was confirmed by anteroposterior (AP) and lateral X-ray. After the entry portal instrumentation was inserted, touching the bone, the entry reamer was passed through it and was inserted into the proximal femur. Then, the reamer assembly and guide pin were removed. (4) Intramedullary reaming: After opening the proximal femur, the ball tip guide rod was inserted, into the ideal positions. Then, the length of the implant was measured and the intramedullary canal was reamed in increments to a size 1–1.5 mm larger than the selected nail size. Using a drill guide handle, the nail was manually advanced into the proximal femur. (5) Integrated interlocking screw insertion: After confirming the position of the main nail, a longitudinal skin incision was made at the entry site of the lag screw, and the lag screw drill sleeve was inserted. Then, the guide pin sleeve was passed through the lag screw drill sleeve until it touched the bone, and a guide pin was inserted into the femoral neck and head. Once the appropriate position of the guide pin was confirmed, the length of the lag screw was measured with a ruler, and a compression screw starter drill was used to drill the lateral cortex. Then, the compression screw drill was passed through the lag screw drill sleeve, inserting into the femoral neck and head, and then the compression screw drill was removed, and an antirotation bar was inserted through the same hole. After the guide pin sleeve was removed, the lag screw drill was then drilled to the measured depth. Then, the lag screw was manually advanced (in compression or no compression mode according to fracture gaps), and the antirotation bar was removed and the compression screw was manually advanced. After the drill guide handle was removed, the nail cap was inserted on the top of the nail. (6) Distal locking: The free-hand technique was used for distal interlocking screw placement. After a stab skin incision was made at the site of screw entry, the short drill was inserted, touching the bone and drilling both cortices. Then, the screw depth gauge was used to measure the length of the screw and a locking screw with the appropriate length was inserted. (7) Closure: After verifying the implant position by both AP and lateral view using the C arm X-ray machine, the incision was closed.

### 2.4. Learning Curve Model

The best general mathematic formulation for a medical learning curve is not known. The negative exponential curve-fit regression analysis has been used to describe the learning curve of radiofrequency ablation of tachyarrhythmias [11] and minimally invasive transforaminal lumbar interbody fusion [12]. The negative exponential curve-fit regression analysis (*Y* = (*Y*0 − Plateau)*∗*exp⁡(−*K∗X*) + Plateau) was used to evaluate the learning curve. *Y* represents the operative time; *X* represents the case number; *Y*0, Plateau, and *Y*0 − Plateau represent the beginner's proficiency level, expert's proficiency level, and potential improvement individually; and *K* is the rate constant. *X*% represents the learning milestone, or the surgery quantity needed to achieve a percentage of potential improvement, is computed as ln⁡(1 − *X*%)/*K*.

### 2.5. Statistical Analysis

Continuous variables, such as operation time, intraoperative blood loss, weight bearing time, Harris score, length of hospital stay, patient age, fracture union time, and TDA, were expressed as means with ranges and then the mean differences between the groups were compared by *t*-test. For categorical variables between the groups, the chi-square test or Fisher exact test was used as appropriate. Data analysis was performed using the GraphPad Prism statistical software (version 5.0), and a value of *p* < 0.05 was considered statistically significant.

## 3. Result

Fifty-three consecutive patients with femoral intertrochanteric fractures (24 right and 29 left) who underwent surgery with InterTan intramedullary nail were included in our study.  Table 1 shows the baseline characteristics of all participants. The participants consisted of 22 males (41.5%) and 31 females (58.5%), with an average age of 80.36 (95% CI 78.41 to 82.31) years. The mean follow-up time was 15.26 (95% CI 14.49 to 15.85) months.

The mean time for hospital stay was 7.79 (95% CI 7.21 to 8.38) days and the mean intraoperative blood loss was 207.25 (95% CI 202.30 to 212.19) ml. The mean time to partial weight bearing was 38.57 (95% CI 37.13 to 40.01) days. The mean time for union was 11.81 (95% CI 11.50 to 12.13) weeks. The mean Harris hip score was 82.15 (95% CI 80.85 to 83.46). The mean tip apex distance (TAD) was 20.80 (95% CI 19.75 to 21.85) mm. There were complications for case numbers: 12, 13, 17, 20, 24, 32, 42, 43, 47, 49, 50, and 54. There were postoperative complications in 12 patients (22.6%) including venous thromboembolism (VTE), pain of hip and thigh, and superficial wound infection, as summarized in Table 2.

### 3.1. Learning Curve of Performing Surgery with InterTan Intramedullary Nail

**Figure 1:**
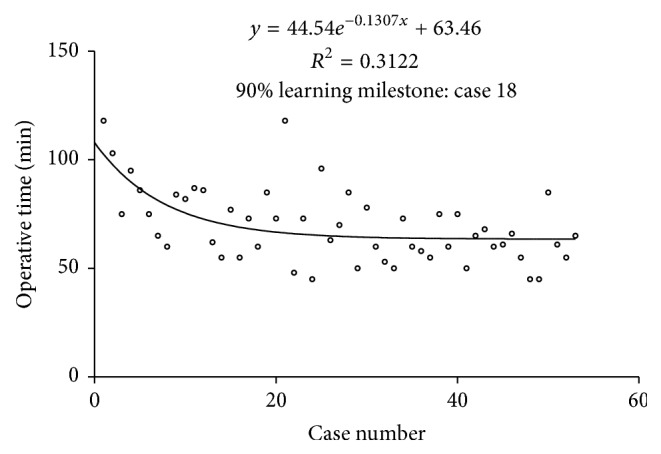
Learning curve of performing surgery with the InterTan intramedullary nail.

The mean operative time was 69.28 (95% CI 64.57 to 74.00) minutes, and with the accumulation of surgical experience, the operation time was gradually decreased (*y* = 44.54*e*^−0.1307*x*^ + 63.46; *R*^2^ = 0.3122; Figure 1). 90% of the potential improvement was expected after 18 cases (90% learning milestone). According to 90% learning milestone, fifty-three were divided into two group; the first 18 cases was early group and others were late group.

### 3.2. Comparison between Early Group (*n* = 18) and Late Group (*n* = 35)

**Figure 2:**
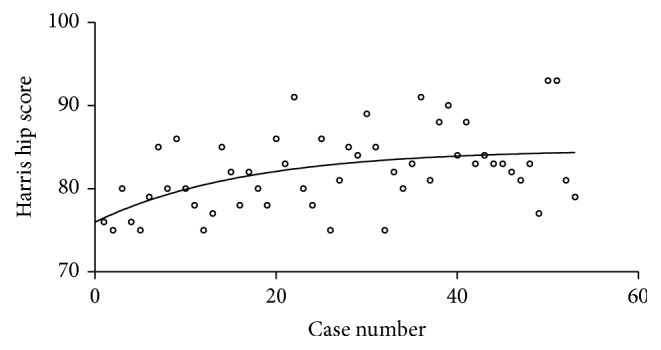
The variation tendency of Harris hip score with the accumulation of surgical cases.

A comparison of the early group and late group showed a significant difference in the operative time, intraoperative blood loss, and hospital stay. In terms of the Harris hip score, significant differences were detected between the two groups (Table 2). With the accumulation of surgical experience, the Harris hip score was gradually increased (Figure 2). Partial weight bearing time, fracture union time, and tip apex distance were similar between two groups (Table 2). There were no significant differences in the number of complications and blood transfusion between the two groups (Table 2).

## 4. Discussion

Various internal fixation devices have been used to treat femoral intertrochanteric fractures, including sliding hip screws (SHS), percutaneous compression plate (PCCP), gamma nail (GN), proximal femora nail (PFN), proximal femoral nail antirotation (PFNA), Proximal Femoral Nail Antirotation-I I (PFNA-II), and the InterTan intramedullary nail. Compared to other implants, the advantages of InterTan nail include the ability to maintain compression, eliminate Z-effect intertrochanteric rotational stability and medial migration, control rotation during reduction, and prevent periprosthetic fractures. Additionally, it has a lower rate of implant failure and reoperation, lower risk of secondary femoral fractures [4, 13–15], faster time to fracture union [14–16], and a high rate of return to prefracture status [2, 14, 15].

To our knowledge, the learning curve of performing surgery with the InterTan intramedullary nail has not been previously studied. Our hypothesis is that surgical experience has significant effects on operative times [17]. The results of our study confirmed our hypothesis and demonstrated that, with the accumulation of surgical experience, the operation time was gradually decreased, and the learning curve for InterTan intramedullary nail was considered acceptable. As shown in Figure 1, the curve declines rapidly in the early phase and gradually reaches a relatively steady state, or asymptote [17].

The mean duration of surgery for InterTan intramedullary nail in the late group of patients was 64.97 (95% CI 59.59 to 70.36) min, similar to the operative time reported in earlier research that ranged from 48 to 73.91 min [4, 5, 15, 18–20], excluding one study that reported an average time that was less than 30 minutes [21]. These studies also reported that the mean operative time for GN, PFNA, or PFNA-II treatments [4, 5, 15, 18–20] was 44.41–72.98 minutes. Of these, two studies reported that there was no significant difference in the operative time between the InterTan intramedullary nail (IT) group and the GN or PFNA group [15, 20]. However, four studies reported that the IT group required a significantly longer surgical time than the GN or PFNA or PFNA-II groups [4, 5, 18, 19], which indicates that InterTan intramedullary nail may increase operative time after we had mastered the technique, although it has some potential clinical advantages, such as increasing Harris hip score, decreasing hospitalization time, and faster time to fracture union.

To a certain extent, with an increased number of surgical cases, surgical technique is gradually improved and the operation time is shortened. With decreased time in surgery, surgical injury and intraoperative bleeding are reduced and effects on the circulatory system are reduced. These factors promote the recovery of patients and reduce the length of hospital stays. This is consistent with our findings that, with the accumulation of experience, the operation time and intraoperative bleeding were gradually decreased, and the hospital stay was also decreased. When comparing a new technique with conventional techniques, it is important that the surgeon should have reached the steady state of the learning curve, indicating that the surgeon acquired proficiency for the new technique [17]. If measured at an earlier point in the learning process, the evaluation of the efficacy of the new technology will be biased. To more accurately compare DHS or PFN or PFNA with IT, surgeons who have mastered the TRIGEN INTERTAN technique by performing more than 18 operations should participate in the surgery group to facilitate an accurate comparison.

We found that the incidence of complications varied considerably in different studies, with ranging from 0% to 19.2% for IT [2, 4, 5, 15, 18–21] and from 2.2% to 34.6% for GN, PFNA, PFNA-II, or DHS [2, 4, 5, 15, 18–21]. In our study, although no serious procedure-related complications were observed, the incidence of complications may be slightly high (22.6%). To compare with GN or PFNA or PFNA-II or DHS, the incidence of complications was not high, and the results were generated when the surgeons were still in the learning phase. Thus, this result can be considered acceptable. In addition, complications showed no significant differences between the two groups. To reduce the incidence of complications, the surgeon will be more accurate during the operation [7], as well as careful treatment and rehabilitation training for patients after surgery in early phase of the learning curve. Increased surgical time during early procedures may have been due to the requirement for extra time to perform the placement as correctly as possible in an attempt to reduce complications. In addition, we compared two groups based on the outcome of the negative exponential function on operating time: 18 cases versus 33 cases. This comparison showed no significant differences in time to partial weight bearing, time for union, or the mean tip apex distance (TAD). The TAD was within the recommended level of <25 mm [22] in both groups in our study

Binder et al. [23] reported that progressive exercise under supervision can significantly promote the rehabilitation of limb function and improve quality of life. As shown in Figure 2, the Harris hip score increased slowly with growing of operative case, suggesting that the improvement of surgical technique is one of the factors that increase the Harris score. However, rehabilitation exercises after operation in elderly patients with hip fracture were more important for the limb function recovery.

This study had some limitations. There are many factors that influence the operative times, including the complexity of fracture. Complicated fracture will inevitably increase the operation time. However, surgeons in the early stages of learning curve usually choose relatively simple cases, which is conducive to improving their self-confidence. This is similar to our study; in our study, complex cases are few which may lead to bias. The bias caused by this choice can only be reduced in the randomized study design. Because our study is a retrospective study, this selection bias is difficult to eliminate. This will inevitably affect the evaluation of the learning curve. It is necessary to carry out a prospective randomized study to reduce the effects of confounding factors such as individual fracture complexity. Although there are many factors that affect the learning curve, it is important to follow the rules of learning curve and realize the key points of study. It is of great significance to carry out a challenging operation in the future. In addition, this report is of the results from a single surgeon and may not be fully applicable to other surgeons. However this study is to provide valuable information and experience for surgeons who decide to learn a new operative method, making them aware of the key learning points and strategies to avoid operative complications. If the learning curve is steep or long, more thorough learning strategies are required, including performing a related exercise on the cadaver model and several cases with an experienced surgeon.

## 5. Conclusions

This study demonstrated that the learning curve of performing surgery with the InterTan intramedullary nail is acceptable and 90% of the expert's proficiency level was achieved at around 18 cases. After surgeons had mastered the technique, the InterTan intramedullary nail could be a reliable and effective option for intertrochanteric fracture.

## Figures and Tables

**Table 1:** Baseline characteristics.

Variable	Early group (*n* = 18)	Later group (*n* = 35)	*p*
Age, mean (95% CI) (years)	80.56 (77.13 to 83.98)	80.26 (77.75 to 82.76)	0.886
Male : female (number)	8 : 10	14 : 21	0.756
Fracture side (right/left) (number)	7 : 11	17 : 18	0.502
Fracture type (AO/OTA) (number)			0.949
A1	5	10	
A2	10	18	
A3	3	7	
Diabetes (number)	2	5	0.75
Hypertension (number)	2	10	0.15
Heart failure (number)	1	0	0.47
Coronary artery disease (number)	1	0	0.16
Chronic cerebral infarction (number)	1	5	0.49
Atrial fibrillation (number)	0	1	0.47
Pulmonary infection (number)	1	4	0.34
ASA score (number)			0.453
1	0	1	
2	13	18	
3	5	15	
4	0	1	

ASA, American Society of Anesthesiologists; CI, confidence interval;

AO/OTA, Arbeitsgemeinschaft für Osteosynthesefragen/Orthopaedic Trauma Association.

**Table 2:** Comparison of clinical outcomes between early group and later group.

Variable	Early group (*n* = 18)	Later group (*n* = 35)	*p*
Time of operation, mean (95% CI) (min)	77.67 (69.18 to 86.15)	64.97 (59.59 to 70.36)	**0.009**
Blood loss, mean (95% CI) (ml)	219.94 (211.44 to 228.45)	200.71 (195.65 to 205.77)	**0.000**
TAD, mean (95% CI) (mm)	21.34 (19.23 to 23.45)	20.52 (19.28 to 21.76)	0.463
Harris hip score, mean (95% CI)	79.39 (77.63 to 81.15)	83.57 (81.96 to 85.15)	**0.002**
Time to union, mean (95% CI) (weeks)	12.11 (11.48 to 12.75)	11.66 (11.29 to 12.02)	0.174
Time to partial weight bearing,	38.22 (35.33 to 41.11)	38.74 (37.03 to 40.45)	0.735
mean (95% CI) (days)			
Hospital stay, mean (95% CI) (days)	8.67 (7.35 to 9.99)	7.34 (6.78 to 7.91)	**0.030**
Transfusion (number)	5	10	0.952
Complication (number)	3	9	0.456
Deep venous thrombosis (number)	0	4	0.136
Pain of hip and thigh (number)	2	3	0.765
Superficial wound infection (number)	1	2	0.981

Values in boldface indicate *p* < 0.05; TAD, tip apex distance; CI, confidence interval.
